# Supplementary material for: Configurational entropy and Adam-Gibbs relation for quantum liquids
Source: Nat Commun. 2026 Apr 9;17:4843. doi: 10.1038/s41467-026-71210-8 (PMC13223219; doi:10.1038/s41467-026-71210-8)
Supplement: Supplementary file 1 — SUPPLEMENTARY INFORMATION [file 41467_2026_71210_MOESM1_ESM.pdf]

Supplementary Information for 'Configurational Entropy and Adam-Gibbs Relation for Quantum Liquids'

Yang Zhou[1,2,*], Ali Eltareb[1,2], Gustavo E. Lopez[3,4], and Nicolas Giovambattista[1,2,3,*]

[1]*Ph.D. Program in Physics, The Graduate Center of the City University of New York, New York, NY 10016, United States*

[2] *Department of Physics, Brooklyn College of the City University of New York, Brooklyn, NY 11210, United States*

[3]*Ph.D. Program in Chemistry, The Graduate Center of the City University of New York, New York, NY 10016, United States*

[4]*Department of Chemistry, Lehman College of the City University of New York, Bronx, NY 10468, United States*

[*]*Corresponding authors: yzhou4@gradcenter.cuny.edu; ngiovambattista@brooklyn.cuny.edu*

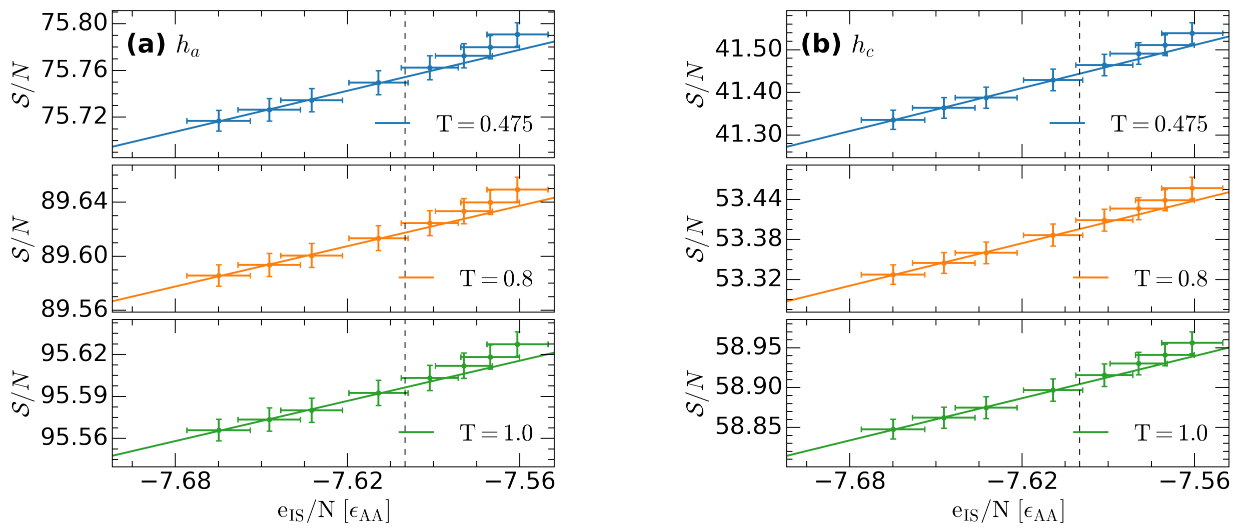

FIG. S1. **Basin shape function of the ring-polymer system.** The shape function is shown as a function of the inherent structure energy $e_{\rm IS}$ for the LJBMs with Planck's constant (a) $h = h_a$ and (b) $h = h_c$. Each panel includes data at three different temperatures. The solid lines are linear fits to the data points for $e_{\rm IS} < -7.6$, where the Gaussian and harmonic approximation of the PEL hold for the classical LJBM. Error bars represent the standard deviation. Source data are provided as a Source Data file.

I. LINEAR DEPENDENCE OF THE SHAPE FUNCTION ON THE INHERENT STRUCTURE ENERGY

In this section, we show that the shape function $\mathcal{S}(N, V, T, e_{\rm IS})$ for the systems studied, defined in Eq. 12 in the main manuscript, is a linear function of the inherent structure energy $e_{\rm IS}$ for all the values of h and T studied. As an example, we include in Fig. S1, the shape function of the ring-polymer system as a function of the inherent structure energy for the LJBMs with Planck's constant h_a and h_c, at temperatures $T = 0.475, 0.8, 1.0$. The shape function at each point is computed from the IS normal mode frequencies of the classical system, using the method described in Ref. [1]. Solid lines are linear fits to the data points with $e_{\rm IS} \leq -7.6$ (dashed line); at $e_{\rm IS} > -7.6$, the Gaussian and harmonic approximation of the PEL no longer hold for the classical LJBM (see also Fig. 2 of the main manuscript). From the linear fits in Fig. S1, we extract the fitting parameters $a(T)$ and $b(T)$ defined in Eq. 30 of the main manuscript.

II. THE IMPORTANCE OF ANHARMONIC CONTRIBUTIONS TO THE CONFIGURATIONAL ENTROPY

The configurational entropy, $S_{\rm IS}(N, V, e_{\rm IS})$, of the classical and quantum LJBMs is given by Eq. 10 (Gaussian approximation) and is shown in Fig. 4(a). In the main manuscript, this expression for $S_{\rm IS}(N, V, e_{\rm IS})$ is validated using Eq. 34 which includes the anharmonic contributions to the Helmholtz free energy, $F_{\rm vib}^{\rm anh}(N, V, T)$. The agreement between Eq. 34 and Eq. 10 is remarkable (see Fig. 5 in the main manuscript).

That anharmonic contributions for the PEL formalism ($F_{\rm vib}^{\rm anh}(N, V, T)$) are relevant for the quantum LJBMs studied is evident from Figs. 2(a) and 2(b) of the main manuscript. One may wonder, how relevant the anharmonic contributions are for the configurational entropy $S_{\rm IS}(N, V, e_{\rm IS})$ given in Eq. 10. To address this point, we test whether Eq. 34 holds when anharmonicities are removed [$F_{\rm vib}^{\rm anh}(N, V, T) \to 0$]. Specifically, here we test whether Eq. 10 satisfies the following expression,

$$S_{\rm IS}(T, e_{\rm IS})/k_B = \ln\left({\rm P}(T, e_{\rm IS})\right) + 3Nn_b \ln(\beta\hbar\omega_0) + \mathcal{S}(T, e_{\rm IS}) + \beta e_{\rm IS} + c(T) \qquad \text{(S1)}$$

As shown in Fig. S2, Eq. S1 is consistent with Eq. 10 for h_a, h_b. However, a close comparison of Fig. S2 and Figs. 2(a)(b) of the main manuscript shows that for $T = 1.0$ (orange symbols), Eq. 34 works slightly better than Eq. S1. As the quantum character of the LJBMs increases, small deviations are observed at lower temperatures. While in Fig. 5(c) of the main manuscript the values of $S_{\rm IS}$ at $T = 0.65$ (blue triangles) obey Eq. 10 (black line), this is not the case when Eq. S1 is used [blue triangles and black line in Fig. S2(c)]. Despite the slightly better performance of Eq. 34, compared to Eq. S1, the anharmonic contributions to the $S_{\rm IS}$ of the LJBMs studied are small for h_a, h_b, h_c, but seem to become increasingly relevant as h further increases ($h > h_c$).

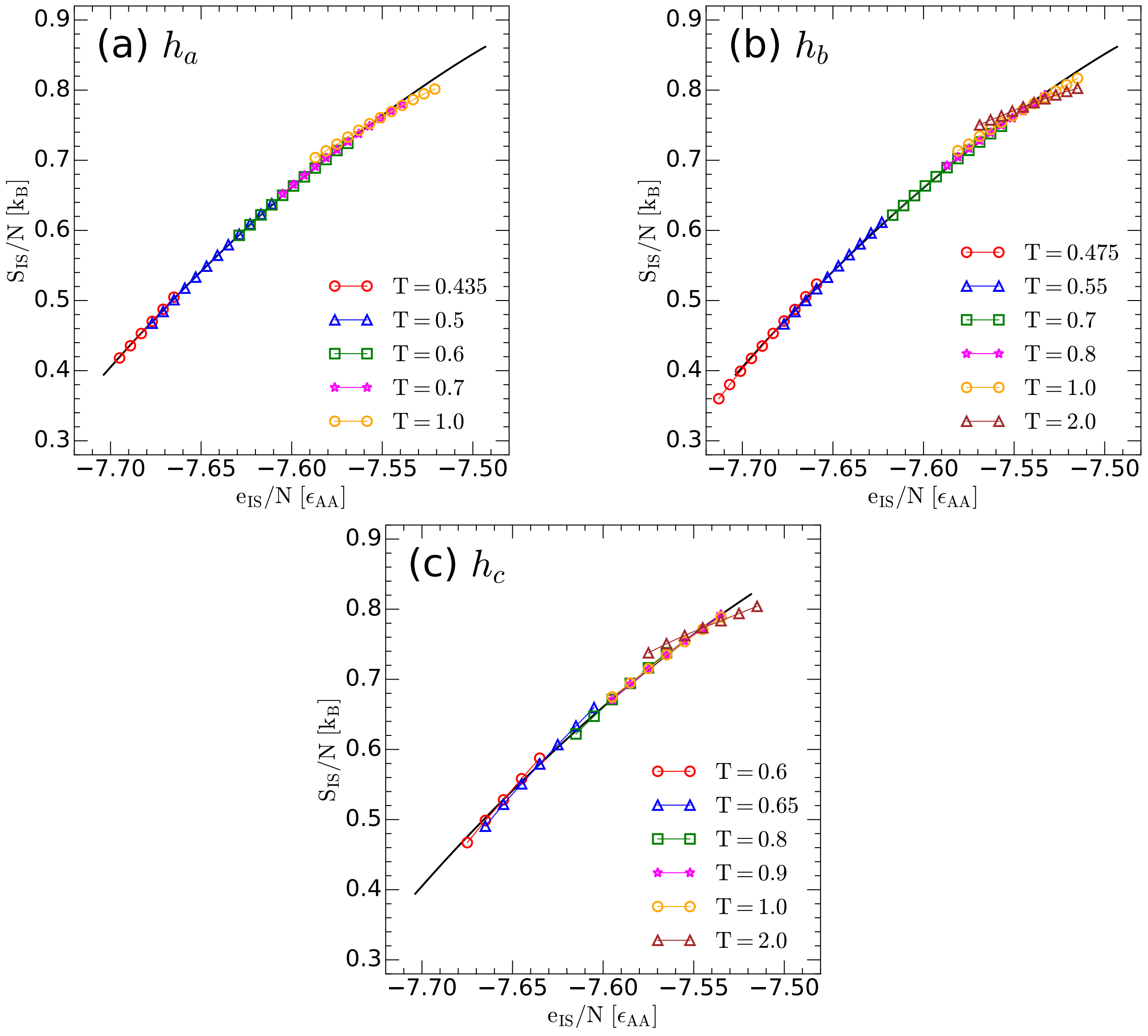

FIG. S2. **Validation of the configurational entropy using the harmonic approximation.** (a) Configurational entropy S_{IS} as a function of the IS energy e_{IS} for the LJBM with $h = h_a$. The solid black line is taken from Fig. 4(a) of the main manuscript; the symbols correspond to the expression on the right-hand-side of Eq. S1 for temperatures $T = 0.435$ (red circles), 0.5 (blue up-triangles), 0.6 (green squares), 0.7 (magenta stars) and 1.0 (orange circles). Results at the different temperatures are obtained from RPMD simulations [datasets are shifted by a constant $c(T)$; see text]. (b) Same as (a) for $h = h_b$, and for temperatures $T = 0.475$ (red circles), 0.55 (blue up-triangles), 0.7 (green squares), 0.8 (magenta stars), 1.0 (orange circles), and 2.0 (maroon up-triangles). (c) Same as (a) for $h = h_c$, and for temperatures $T = 0.6$ (red circles), 0.65 (blue up-triangles), 0.8 (green squares), 0.9 (magenta stars), 1.0 (orange circles), and 2.0 (maroon up-triangles). In contrast to Fig. 5 of the main manuscript, the orange symbols in (a) and (b) [T=1.0] deviate slightly from the black line. Similarly, the blue triangles in (c) [T=0.65] deviate slightly from the black line. Source data are provided as a Source Data file.

III. ENTROPY OF THE CLASSICAL AND QUANTUM LJBM

The total entropy for the studied LJBMs as a function of temperature $S(T)$ for $V = 9.4$ is obtained by thermodynamic integration as performed in Refs. [5, 6] for the classical LJBM. Briefly, for each of the classical/quantum LJBMs considered, characterized by a given value of h, we follow the two-step process detailed below.

(i) In the first step, we evaluate the entropy of the target LJBM at the reference state $T_0 = 5.0$ and $V_0 = 9.4$ (in reduced units); V_0 is the volume studied in the main manuscript. To do so, we compress the LJBM from a very large volume $V_{\rm ig} = 3\times10^5$ to the target volume V_0 along the isotherm, $T_0 = 5.0$. From the thermodynamic relationship $dE = T\,dS - P\,dV$, one obtains the following expression,

$$S(T_0, V_0) = S(T_0, V_{\rm ig}) + \frac{1}{T_0}\left[E(T_0, V_0) - E(T_0, V_{\rm ig})\right] - \frac{1}{T_0}\int_{V_0}^{V_{\rm ig}} P(T_0, V)\,dV \qquad \text{(S2)}$$

The volume $V_{\rm ig}$ is very large and the temperature T_0 is very high for all the LJBMs considered; at these conditions, $h/\sqrt{2\pi m_A k_B T_0} \ll V_{\rm ig}^{1/3}$ for all the values of h studied. Accordingly, at $(T_0, V_{\rm ig})$, the (quantum and classical) LJBM can be approximated by an ideal gas binary mixture. Hence, $E(T_0, V_{\rm ig})$ and $S(T_0, V_{\rm ig})$ in Eq. S2 are given by the energy and entropy of the ideal gas binary mixture, i.e., $E(T_0, V_{\rm ig}) = E_{\rm ig}(T_0, V_{\rm ig}) = \frac{3}{2}Nk_BT_0$ (where $N = N_A + N_B$) and $S(T_0, V_{\rm ig}) = S_{\rm ig}(T_0, V_{\rm ig})$; the same expressions hold for both the quantum and classical ideal gas binary mixtures since $h/\sqrt{2\pi m_A k_B T_0} \ll V_{\rm ig}^{1/3}$ [3, 7]. The entropy of a (classical or quantum) ideal gas binary mixture at (T, V) is given by $(m_A = m_B)$ [6],

$$\frac{S_{\rm ig}(T, V)}{Nk_B} = -\frac{N_A}{N}\ln\left(\frac{N_A}{N}\right) - \frac{N_B}{N}\ln\left(\frac{N_B}{N}\right) + \frac{3}{2}\ln\left(\frac{2\pi m_A V^{\frac{2}{3}}}{\beta h^2}\right) - \ln\left(\frac{N}{e^{\frac{5}{2}}}\right) \qquad \text{(S3)}$$

In this expression, all quantities are given in real units. Accordingly, to obtain $S_{\rm ig}(T_0, V_0)$ we substitute $T = T_0 \times (\epsilon_{\rm AA}/k_B)$ and $V = V_0 \times (\sigma_{\rm AA}^3)$ in Eq. S3 (the reduced units of T and V are $[T] = \epsilon_{\rm AA}/k_B$ and $[V] = \sigma_{\rm AA}^3$). For the classical LJBM, $h = 6.62607015 \times 10^{-34}$ J s in Eq. S3. For the quantum LJBMs characterized by Planck's constant $h_a = 0.2474$, $h_b = 0.5000$ and $h_c = 1.0000$ in reduced units, $[h] = \sigma_{\rm AA}(m_A\epsilon_{\rm AA})^{1/2}$, the value of h in Eq. S3 is given by $h = h_x \times [\sigma_{\rm AA}(m_A\epsilon_{\rm AA})^{1/2}]$ (where $x = a, b, c$). Note that the right-hand-side of Eq. S3 depends only on h_x and is independent of the specific values of $(m_A, \sigma_{\rm AA}, \epsilon_{\rm AA})$ considered. The values of $S_{\rm ig}(T_0, V_{\rm ig})/Nk_B$ obtained from Eq. S3 are included in Table S1.

System	h $[Js]$	$S_{\text{ig}}(T_0, V_{\text{ig}})/Nk_B$	$E(T_0, V_0)/N$ $[\epsilon_{\text{AA}}]$	$S(T_0, V_0)/Nk_B$
Classical	6.62607×10^{-34}	22.1628	5.8399	13.5865
$h_a = 0.2474$	2.59669×10^{-33}	18.0619	5.8759	9.5120
$h_b = 0.5000$	5.24796×10^{-33}	15.9512	6.0542	7.4084
$h_c = 1.0000$	1.04959×10^{-32}	13.8718	7.0975	5.4021

TABLE S1. **Reference state point parameters for the LJBMs studied.** Values of the Planck's constant studied in the main manuscript given in reduced units (first column). For comparison, included in the second column are the corresponding values of h in real units, obtained from the first column via the expression $h = h_x \times [\sigma_{\text{AA}}(m_A \epsilon_{\text{AA}})^{1/2}]$ and assuming ($m_A = 39.948$ amu, $\sigma_{\text{AA}} = 1.0$ Å, $\epsilon_{\text{AA}} = 1.0$ kJ/mol). $S_{\text{ig}}(T_0, V_{\text{ig}})$ is the entropy of the ideal gas binary mixture given by Eq. S3 at $T_0 = 5.0$ and $V_{\text{ig}} = 3\times10^5$ (both quantities given in reduced units). $E(T_0, V_0)$ and $S(T_0, V_0)$ are the energy and entropy of the LJBMs studied at the reference state point ($T_0, V_0 = 9.4$); $S(T_0, V_0)$ is evaluated using Eq. S2.

To evaluate Eq. S2, we perform additional MD/PI computer simulations at $T = T_0$ and different values of V in the range $[V_0, V_{\text{ig}}]$. Fig. S3 shows the excess pressure $P_{\text{ex}}(T_0, V) = P(T_0, V) - Nk_BT_0/V$ of the LJBMs obtained from the MD/PI computer simulations. The values of $E(T_0, V_0)$ and $S(T_0, V_0)$ [Eq. S2] are given in Table S1.

(ii) The second step to get $S(T, V_0)$ consists of a thermodynamic integration along the V_0-isochore starting from the reference temperature T_0. Specifically, since $dE = T\,dS$ along an isochore, one obtains the following expression,

$$S(T) = S(T_0) - \int_T^{T_0} \frac{C_v(T')}{T'}\, dT' \qquad \text{(S4)}$$

where $C_v(T) = \left(\frac{\partial E}{\partial T}\right)_V$ is the constant-volume heat capacity (for simplicity, from now on, we omit the dependence of all quantities on V_0). To evaluate $C_v(T)$, we perform MD/PI computer simulations at various temperatures ($V = V_0$) and calculate the total energy of the quantum LJBM, $E(T)$. For the classical case, we follow Refs. [5, 6] and use Tarazona's approximation [4] for the potential energy, $U(T) = a_0 + b_0T^{5/3}$ (where a_0 and b_0 are constants). Accordingly, for the classical LJBM, we interpolate $E(T)$ using the expression for the total energy $E(T) = \frac{3}{2}Nk_BT + a_0 + b_0T^{5/3}$. For the quantum LJBMs, the values of $E(T)$ cannot be fitted using this approach. In these cases, $E(T)$ is fitted using a fifth-order poly-

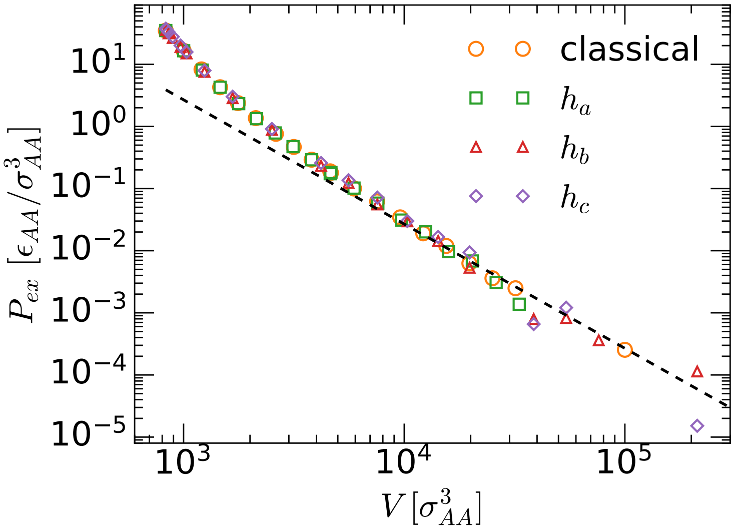

FIG. S3. **Excess pressure of the LJBMs.** Excess pressure $P_{\text{ex}}(T_0, V)$ of the LJBMs obtained from MD/PI computer simulations at $T_0 = 5.0$ and $V \geq V_0 = 9.4$. The dashed line is the first virial correction to the pressure calculated analytically, $B_2(T_0)k_BT_0\,(N/V)^2$, with $B_2(T_0) = 0.53622$ (see Ref. [6]). Source data are provided as a Source Data file.

nomial $E(T) = \sum_{i=0}^{5} a_i T^i$. Fig. S4(a) shows the $E(T)$ obtained from the MD/PI computer simulations together with the corresponding fifth-order polynomial fit. The entropy $S(T)$ is evaluated analytically using Eq. S4 and is included in Fig. S4(b).

In Fig. S4 (and Fig. 2 (c)(d) of the manuscript), the temperature $T = 0.55$ for $h = h_c$ (purple lines) was not included in the fitting because, at this temperature, the values of $D(T)$ [Fig. 1(c)] and $E_{\text{IS}}(T)$ [Fig. 3(a)] deviate from the corresponding trend observed at higher T ($h = h_c$), probably due to poor equilibration. Nonetheless, even for ($h = h_c$, $T = 0.55$) (purple line in Fig. S4), we find that the entropy is positive, $S = 0.225$. Summarizing, as shown in Fig. S4, $S(T) > 0$ for all the LJBMs studied (as expected). We confirm that the results here are not sensitive to alternative (reasonable) fits to $E(T)$.

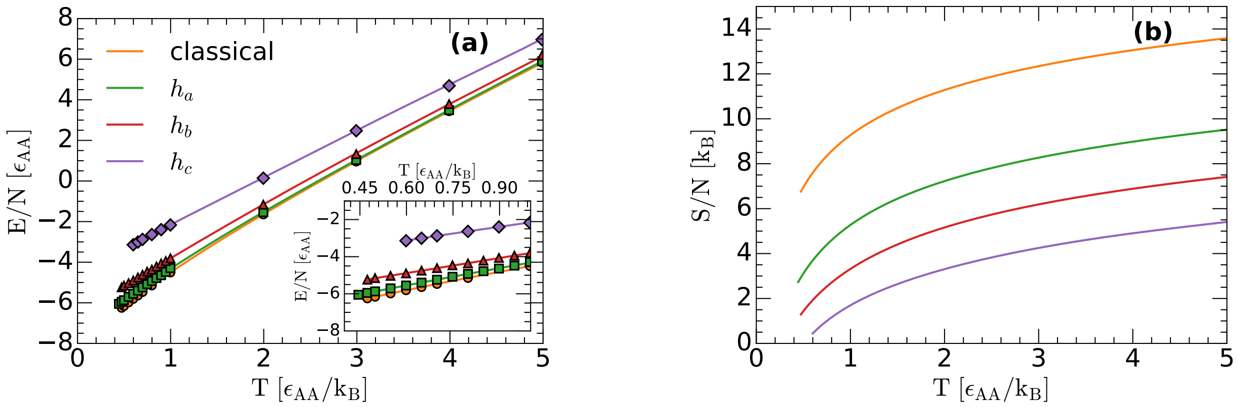

FIG. S4. **Energy and entropy of the LJBMs from thermodynamic integration.** (a) Energy of the classical and quantum LJBMs as a function of temperature ($V = 9.4$) obtained from MD and PI computer simulations with different values of Planck's constant h (symbols). Lines are the polynomial fittings to the symbols [see text]. (b) Entropy of the LJBMs obtained from (a) based on thermodynamic integration, Eq. S4. Source data are provided as a Source Data file.

IV. CONFIGURATIONAL ENTROPY

In this section, we discuss how the configurational entropy $S_{\mathrm{IS}}(T)$ of the classical and quantum LJBMs is obtained numerically, using MD/PI computer simulations and thermodynamic integration. As shown in Fig. 4(b) of the main manuscript, for the quantum LJBMs, the values of $S_{\mathrm{IS}}(T)$ evaluated numerically (symbols) are in very good agreement with the corresponding predictions from the PEL formalism [Eqs. 10 and 28] (solid lines).

The numerical procedure to evaluate $S_{\mathrm{IS}}(T)$ based on thermodynamic integration has been used in the past to calculate the configurational entropy of classical atomistic and molecular liquids, including the classical LJBM [5, 6] and water [1, 2]. This numerical approach is based on the concept of vibrational entropy, S_{vib}, defined implicitly via the expression

$$S(N,V,T) = S_{\mathrm{IS}}(N,V,T) + S_{\mathrm{vib}}(N,V,T) \tag{S5}$$

To evaluate $S_{\mathrm{IS}}(T)$, one calculates $S(T)$ and $S_{\mathrm{vib}}(T)$ independently. The calculation of $S(T)$ for the classical and quantum LJBMs studied is performed by thermodynamic integration, as explained in Sec. III above. To calculate $S_{\mathrm{vib}}(T)$, one evaluates the corresponding expression from the PEL formalism using MD/PI simulations, as explained next.

In the PEL formalism (for a fixed composition, $N_A = 4\,N_B$ and $N = N_A + N_B$), the Helmholtz free energy can be written as

$$F(N,V,T) = F_{\mathrm{IS}}(N,V,T) + F_{\mathrm{vib}}(N,V,T), \tag{S6}$$

where $F_{\mathrm{IS}}(N,V,T) \equiv E_{\mathrm{IS}}(N,V,T) - T\,S_{\mathrm{IS}}(N,V,E_{\mathrm{IS}})$ [see Eq. 13 of the main manuscript]. Since the Helmholtz free energy is $F(N,V,T) = E(N,V,T) - T\,S(N,V,T)$ and the vibrational energy is defined such that $E(N,V,T) \equiv E_{\mathrm{IS}}(N,V,T) + E_{\mathrm{vib}}(N,V,T)$, it follows from Eqs. S5 and S6 that

$$S_{\mathrm{vib}}(N,V,T) = \frac{E_{\mathrm{vib}}(N,V,T) - F_{\mathrm{vib}}(N,V,T)}{T}. \tag{S7}$$

(i) Classical LJBM. Following Refs. [5, 6], for the classical LJBM, it can be shown that Eq. S7 leads to the expression

$$S_{\mathrm{vib}}(N,V,T) = 3Nn_b k_B\left[1 - \ln(\beta\,\hbar\,\omega_0)\right] - k_B\,\mathcal{S}(N,V,T,e_{\mathrm{IS}}). \tag{S8}$$

Expression S8 assumes that the PEL of the classical LJBM is Gaussian and harmonic, which is consistent with MD simulations [5, 6] (see also Figs. 2(a) and 2(b) of the main manuscript).

Our values of $S_{\rm IS}(T)$ for the classical LJBM, obtained numerically using Eqs. S5 and S8, are shown in Fig. S5(a).

Following the procedure in Ref. [6], from the $S_{\rm IS}(T)$ shown in Fig. S5(a), we obtain $S_{\rm IS}(e_{\rm IS})$. The symbols in Fig. S5(b) correspond to the resulting $S_{\rm IS}(e_{\rm IS})$ evaluated at selective values of $e_{\rm IS}$. The line in Fig. S5(b) is the prediction from the PEL formalism based on the Gaussian approximation, Eq. 10. Following Ref. [5], in the classical case, we obtain E_0 and σ^2 from the fit in Fig. 2(a) [orange line] based on Eq. 15; the parameter α is fit to maximize the overlap of the numerical values of $S_{\rm IS}(e_{\rm IS})$ (symbols) and the solid line in Fig. S5(b) [Eq. 10].

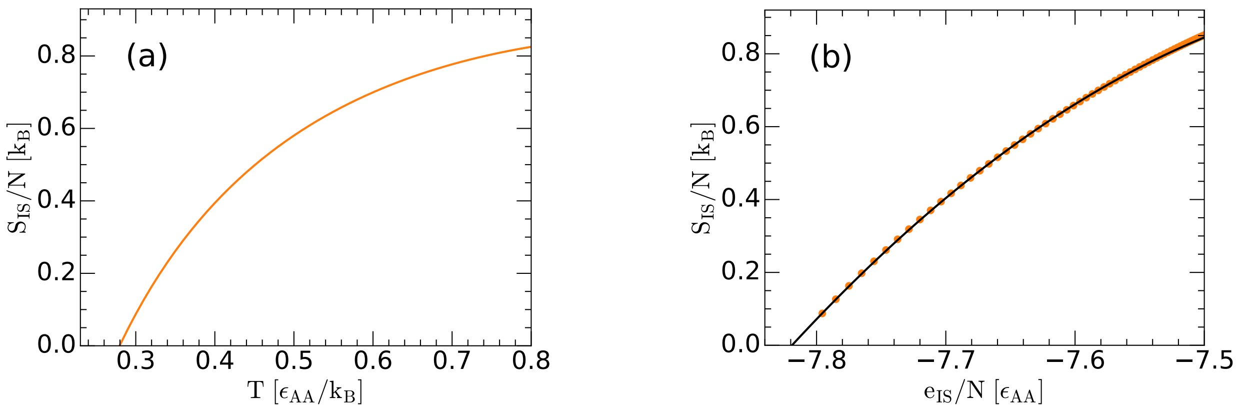

FIG. S5. **Configurational entropy of the classical LJBM.** (a) Configurational entropy $S_{\rm IS}(T)$ of the classical LJBM as a function of temperature. $S_{\rm IS}(T)$ is calculated numerically following the procedure of Refs. [5, 6] via thermodynamic integration and MD simulations. (b) $S_{\rm IS}(e_{\rm IS})$ obtained from (a) for selected values of $e_{\rm IS}$ (symbols); see Ref. [6]. The line is the prediction from the PEL formalism based on the Gaussian approximation, Eq. 10. In Eq. 10 the parameters E_0 and σ^2 are obtained from Fig. 2(a) using Eq. 15; the parameter α is fit to maximize the overlap of the numerical values of $S_{\rm IS}(e_{\rm IS})$ (symbols) and the solid line [Eq. 10]. We obtain $E_0 = 7304.012$, $\sigma^2 = 134.223$, $\alpha = 0.988$. Source data are provided as a Source Data file.

(ii) Quantum LJBMs. Using Eqs. 27 and 29 of the main manuscript in Eq. S7 leads to

the following expression for the quantum LJBMs,

$$\begin{aligned} S_{\rm vib}(N,V,T) &= 3Nn_bk_B\left[1-\ln(\beta\,\hbar\,\omega_0)\right] \\ &- k_B\left[\mathcal{S}(N,V,T,E_{\rm IS})+\tilde{B}_0(N,V,T)+\tilde{B}_1(N,V,T)\,E_{\rm IS}(N,V,T)\right] \\ &+\frac{1}{T}\left[\left(\frac{\partial\,\mathcal{S}}{\partial\beta}\right)_{N,V,E_{\rm IS}}+\left(\frac{\partial\tilde{B}_0}{\partial\beta}\right)_{N,V}+\left(\frac{\partial\tilde{B}_1}{\partial\beta}\right)_{N,V}E_{\rm IS}(N,V,T)\right]. \end{aligned} \tag{S9}$$

Most of the quantities needed in Eq. S9 (specifically, the PEL variables $\{\alpha, E_0, \sigma^2, a, b, c_{0,1}, c_{0,2}, c_{1,0}, c_{1,1}, c_{1,2}\}$) are reported in the main manuscript. The only missing quantity in Eq. S9 is $c_{0,0}$. Therefore, using Eqs. S5 and S9, one can evaluate $S_{\rm IS}(T)$ up to the unknown (additive) T-independent quantity $c_{0,0}$. The symbols in Fig. 4(b) of the main manuscript are the values of $S_{\rm IS}(T)$ for the quantum LJBMs obtained numerically following the procedure described here. For each value of h, the additive constant $c_{0,0}$ (for fixed $V = 9.4$) is fit to maximize the overlap with the PEL predictions (lines in Fig. 4(b)). The agreement with the theoretical predictions based on the PEL formalism is very good. For comparison, Figs. S6(a)–(c) show the same values of $S_{\rm IS}(T)$ included in Fig. 4(b) of the main manuscript but expressed as a function of $e_{\rm IS}$, using Eq. 28 (symbols). The agreement in Figs. S6(a)–(c) between the numerical values of $S_{\rm IS}(e_{\rm IS})$ (symbols) and the PEL predictions based on the Gaussian approximation, Eq. 10 (lines), is very good for all the quantum LJBM studied.

The vibrational contributions to the total entropy for the LJBMs studied, $S_{\rm vib}(T)$, are shown in Fig. S7. For all the LJBMs studied, $S_{\rm vib}(T)$ decreases monotonically upon cooling. Curiously, while $S_{\rm vib}(T) > 0$ for the classical LJBM and quantum LJBM with $h = h_a$ and h_b, for the most quantum LJBM ($h = h_c$), we find that $S_{\rm vib}(T)$ becomes slightly negative at the lowest temperatures accessible in our PI simulations, at approximately $T < 0.65$. We note, however, that $S_{\rm vib}(T)$ is a quantity defined within the PEL formalism with no direct physical interpretation. Indeed, $S_{\rm vib}(T) \equiv S(T) - S_{\rm IS}(T)$ and hence it is the difference between two positive quantities. In the limit $T \to 0$, $S(T) \to 0$ but $S_{\rm IS}(T)$ may remain positive. This can be shown by considering a single quantum atom (ring-polymer) in a symmetric double-well potential with a finite energy barrier. In this case, $S(T) \to 0$ as $T \to 0$ but $S_{\rm IS}(T) = k_B \log 2$, resulting in $S_{\rm vib}(T) < 0$ at low temperatures.

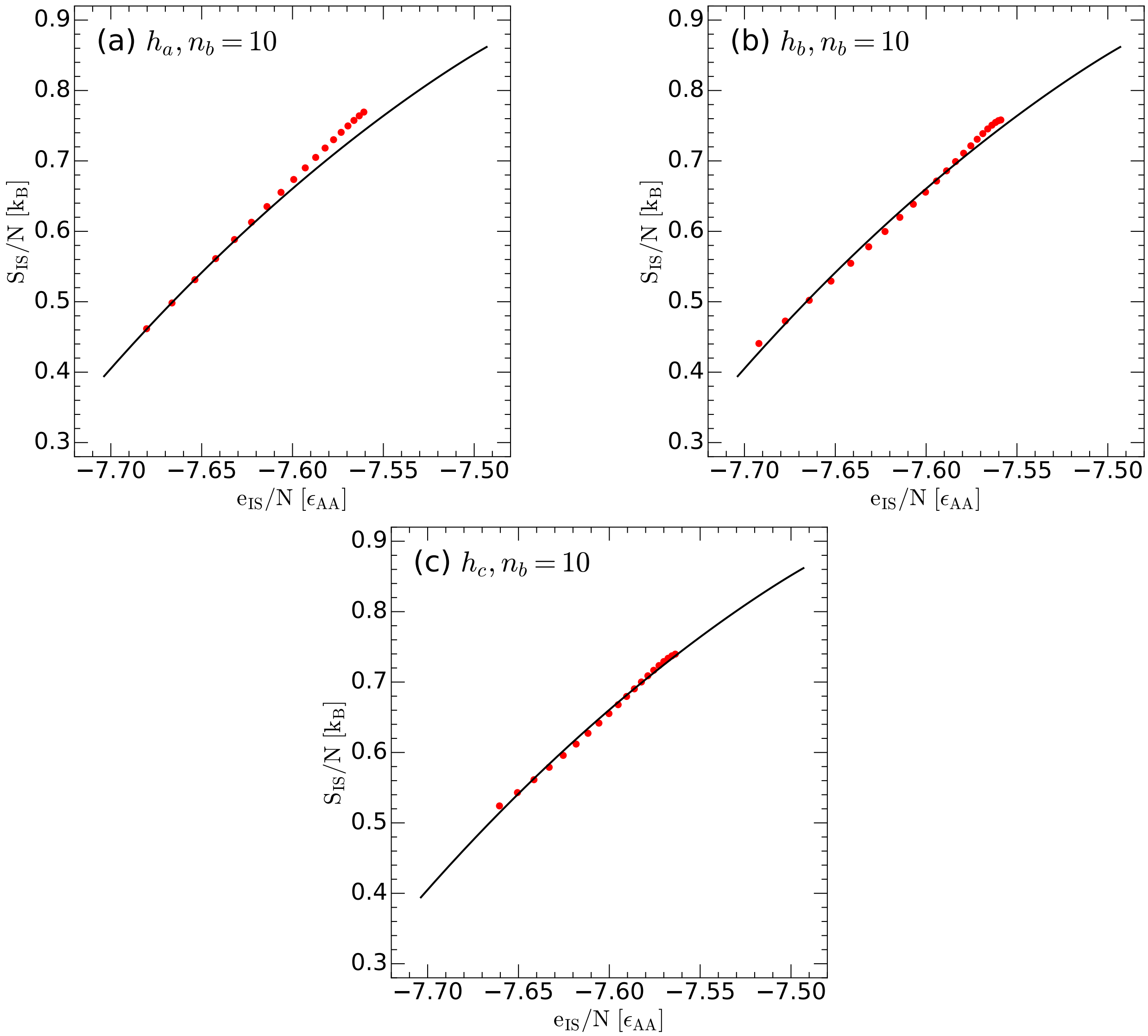

FIG. S6. **Configurational entropy of the quantum LJBMs.** (a) Configurational entropy as a function of the IS energy, $S_{IS}(e_{IS})$, for the quantum LJBMs with Planck's constant (a) $h = h_a$, (b) $h = h_b$, and (c) $h = h_c$. Symbols are the values of $S_{IS}(e_{IS})$ obtained numerically, based on Eqs. S5 and S9, and thermodynamic integration. The line is the expression for $S_{IS}(e_{IS})$ based on the Gaussian approximation of the PEL, Eq. 10, taken from Fig. S5(b). Source data are provided as a Source Data file.

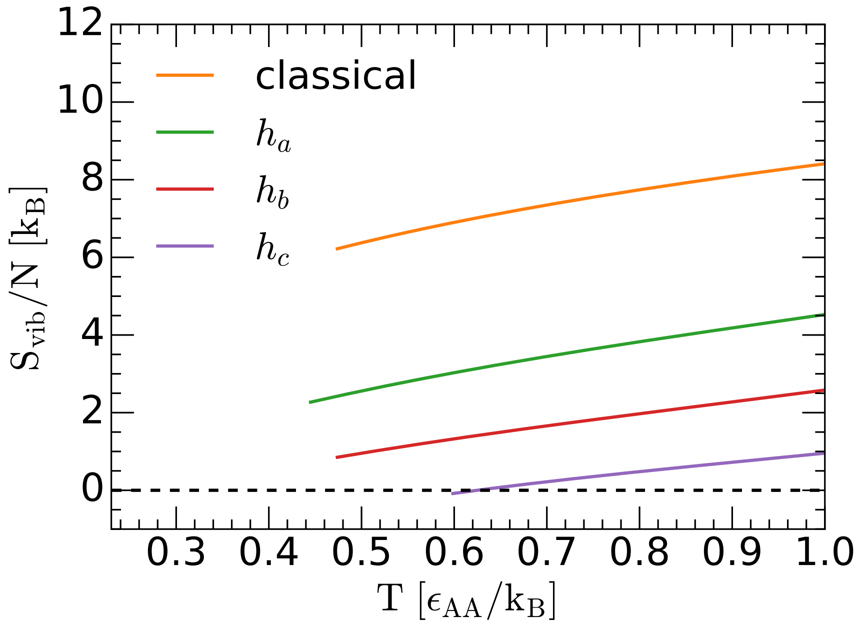

FIG. S7. **Vibrational entropy of the LJBMs.** Vibrational entropy $S_{\rm vib}(T) \equiv S(T) - S_{\rm IS}(T)$ associated to the classical and quantum LJBMs studied with $h = h_a, h_b, h_c$. $S_{\rm vib}(T)$ decreases monotonically upon cooling and remains positive except for the most quantum LJBM studied ($h = h_c$; purple line) at very low temperatures (approximately $T < 0.65$). Source data are provided as a Source Data file.

V. TEMPERATURE-DEPENDENCE OF THE DIFFUSION COEFFICIENT

The diffusion coefficient $D(T)$ of the A-type particles in the classical and quantum LJBMs studied are shown in Fig. 1(c) of the manuscript. As shown in Fig. S8, the $D(T)$ of the LJBMs obey the Vogel-Fulcher-Tammann (VFT) equation,

$$D(T) = D_0 \exp\left(-\frac{A}{T - T_{\mathrm{VFT}}}\right) \tag{S10}$$

where A is a constant and T_{VFT} is the VFT temperature. T_{VFT} indicates approximately the temperature at which $D(T) = 0$ and hence, it is a good estimation of the glass transition temperature of the system.

The values of $T_{\mathrm{VFT}}(h)$ for all the LJBMs studied are included in Fig. S8(b) together with the corresponding values of Kauzmann temperature $T_K(h)$ reported in the main manuscript. The values of $T_{\mathrm{VFT}}(h)$ and $T_K(h)$ are remarkably close to one another; both $T_{\mathrm{VFT}}(h)$ and $T_K(h)$ increase approximately linearly with increasing quantumness (h), for the values of h studied here. However, as shown in [8], further increase in h (for $h > h_c$) leads to a change in the dynamics of the LJBM where the $D(T)$ increases with increasing h. Accordingly, both $T_{\mathrm{VFT}}(h)$ and $T_K(h)$ are expected to decrease with increasing h, for $h > h_c$.

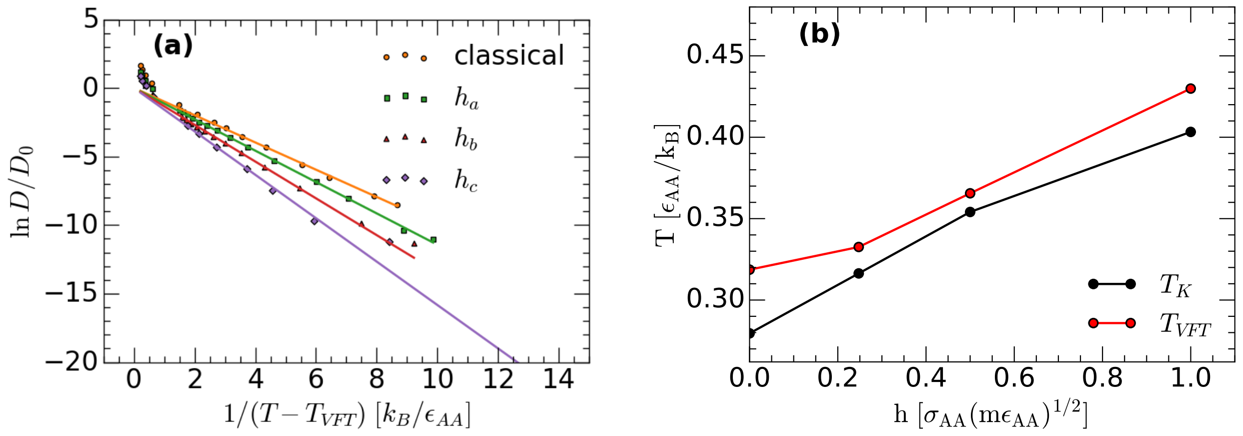

FIG. S8. **Diffusion coefficient of the LJBMs.** (a) Diffusion coefficient as a function of temperature for the classical and quantum LJBMs studied. Lines are fits using the VFT equation, Eq. S10. (b) VFT temperature $T_{\rm VFT}(h)$ obtained from the fits in (a). For comparison, the Kauzmann temperature $T_K(h)$ is also included. The values at $h = 0$ are for the classical LJBM (MD simulations). Both $T_{\rm VFT}(h)$ and $T_K(h)$ exhibit the same qualitative behavior. Source data are provided as a Source Data file.

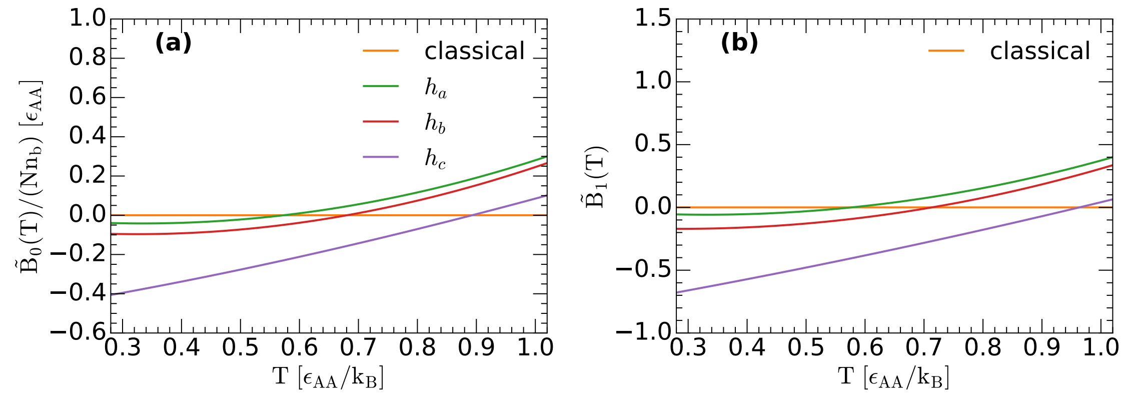

FIG. S9. **Anharmonic contributions for the LJBMs.** Coefficient $\tilde{B}_0$ and $\tilde{B}_1$ defined in Eq. 24 and obtained from the fittings in Figs. 2(c)(d). Source data are provided as a Source Data file.

VI. INHERENT STRUCTURE ENERGY ($E_{\rm IS}$) AND VIBRATIONAL ENERGY ($E_{\rm vib}$) FOR A GAUSSIAN POTENTIAL ENERGY LANDSCAPE

Here, we show how the formal expressions for $E_{\rm IS}(N,V,T)$ and $E_{\rm vib}(N,V,T)$ are obtained in the PEL formalism. The PEL is assumed to be Gaussian. We consider both cases where the harmonic approximation of the PEL holds (Eqs. 15 and 16), and where anharmonicities are included (Eqs. 18 and 20).

Harmonic PEL. The expression for $E_{\rm IS}$ (Eq. 15) follows from Eq. 8, using the expression for the configurational entropy of a Gaussian PEL (Eq. 10) and the vibrational Helmholtz free energy for a harmonic PEL (Eq. 11). Specifically, substituting Eqs. 10 and 11 into Eq. 8, one can show that,

$$1 + k_BT\left(\frac{e_{\rm IS}-E_0}{\sigma^2}\right) + k_BT\left(\frac{\partial \mathcal{S}}{\partial e_{\rm IS}}\right)_{N,V,T} = 0 \quad \text{at } e_{\rm IS} = E_{\rm IS}. \tag{S11}$$

It follows that,

$$E_{\rm IS}(N,V,T) = E_0(V) - \sigma^2(V)\left(\beta + b(N,V,T,E_{\rm IS})\right) \tag{S12}$$

where $b(N,V,T,e_{\rm IS}) \equiv \left(\frac{\partial \mathcal{S}(N,V,T,e_{\rm IS})}{\partial e_{\rm IS}}\right)_{N,V,T}$. Eq. S12 defines $E_{\rm IS}$ implicitly. However, for most systems studied, including the LJBMs studied in this work, it is found that $b = b(N,V,T)$ [see Eq. 30] and hence, Eq. S12 can be used to define $E_{\rm IS}$ explicitly. To do so, one first needs to calculate $\mathcal{S}(N,V,T,e_{\rm IS})$, from which $b(N,V,T)$ is derived. Since the PEL variables E_0 and σ^2 are assumed to be T-independent (i.e., $E_0 = E_0(V)$ and $\sigma^2 = \sigma^2(V)$), these variables can be obtained by plotting $E_{\rm IS}(T)$ as a function of $\beta + b$, and fitting the data using Eq. S12 [see, e.g., Fig. 3(a)].

The expression for $E_{\rm vib}$ (Eq. 16) follows from the definition $E_{\rm vib} \equiv E - E_{\rm IS}$ and the thermodynamic expression $E = \left(\frac{\partial(\beta F)}{\partial \beta}\right)_{N,V}$. Since $F = -k_BT\ln Q$, it follows from Eq. 7 that,

$$E(N,V,T) = \left(\frac{\partial}{\partial \beta}\left[\beta E_{\rm IS} - \frac{1}{k_B}S_{\rm IS} + \beta F_{\rm vib}\right]\right)_{N,V} \tag{S13}$$

The first two terms of Eq. S13 can be calculated using Eq. 10 (Gaussian approximation),

$$\left(\frac{\partial}{\partial \beta}\left[\beta E_{\rm IS} - \frac{1}{k_B}S_{\rm IS}\right]\right)_{N,V} = \left(\frac{\partial}{\partial \beta}\left[\beta E_{\rm IS} - \left(\alpha N - \frac{(E_{\rm IS}-E_0)^2}{2\sigma^2}\right)\right]\right)_{N,V} \tag{S14}$$

Using Eq. S12 in Eq. S14, one obtains the following expression,

$$\left(\frac{\partial}{\partial\beta}\left[\beta E_{\mathrm{IS}}-\frac{1}{k_B}S_{\mathrm{IS}}\right]\right)_{N,V}=E_{\mathrm{IS}}-N\left(\frac{\partial\alpha}{\partial\beta}\right)_V+\beta\left(\frac{\partial E_0}{\partial\beta}\right)_V+\frac{1}{2}\left(\frac{\partial\sigma^2}{\partial\beta}\right)_V(b^2-\beta^2)+b\sigma^2\left[1+\left(\frac{\partial b}{\partial\beta}\right)_{N,V}\right] \tag{S15}$$

The last term of Eq. S13 can be calculated using Eq. 11 (harmonic approximation),

$$\left(\frac{\partial(\beta F_{\mathrm{vib}})}{\partial\beta}\right)_{N,V}=3Nn_bk_BT+\left[\frac{\partial\mathcal{S}(N,V,T,E_{\mathrm{IS}})}{\partial\beta}\right]_{N,V} \tag{S16}$$

Since $\mathcal{S}=\mathcal{S}(N,V,T,E_{\mathrm{IS}})$, it follows that

$$\begin{aligned}\left(\frac{\partial\mathcal{S}}{\partial\beta}\right)_{N,V}&=\left(\frac{\partial\mathcal{S}}{\partial\beta}\right)_{N,V,E_{\mathrm{IS}}}+\left(\frac{\partial\mathcal{S}}{\partial E_{\mathrm{IS}}}\right)_{N,V,\beta}\left(\frac{\partial E_{\mathrm{IS}}}{\partial\beta}\right)_{N,V}\\&=\left(\frac{\partial\mathcal{S}}{\partial\beta}\right)_{N,V,E_{\mathrm{IS}}}+b\left(\frac{\partial E_{\mathrm{IS}}}{\partial\beta}\right)_{N,V}\end{aligned} \tag{S17}$$

Using Eq. S12 in Eq. S17, and then replacing the result into Eq. S15, one finds that

$$\left(\frac{\partial(\beta F_{\mathrm{vib}})}{\partial\beta}\right)_{N,V}=3Nn_bk_BT+\left(\frac{\partial\mathcal{S}}{\partial\beta}\right)_{N,V,E_{\mathrm{IS}}}+b\left[\left(\frac{\partial E_0}{\partial\beta}\right)_V-\sigma^2\left(\frac{\partial}{\partial\beta}(\beta+b)\right)_{N,V}-\left(\frac{\partial\sigma^2}{\partial\beta}\right)_V(\beta+b)\right] \tag{S18}$$

Using Eqs. S15 and S18 in Eq. S13, the following expression for the energy follows,

$$E(N,V,T)=E_{\mathrm{IS}}+\left\{3Nn_bk_BT+\left(\frac{\partial\mathcal{S}}{\partial\beta}\right)_{N,V,E_{\mathrm{IS}}}-N\left(\frac{\partial\alpha}{\partial\beta}\right)_V+(b+\beta)\left(\frac{\partial E_0}{\partial\beta}\right)_V-\frac{1}{2}(b+\beta)^2\left(\frac{\partial\sigma^2}{\partial\beta}\right)_V\right\} \tag{S19}$$

where the curly bracket is $E_{\mathrm{vib}}\equiv E-E_{\mathrm{IS}}$. For the case where E_0, σ^2, and α are T-independent, one finds that

$$E_{\mathrm{vib}}(N,V,T)=3Nn_bk_BT+\left(\frac{\partial\mathcal{S}(N,V,T,E_{\mathrm{IS}})}{\partial\beta}\right)_{N,V,E_{\mathrm{IS}}} \tag{S20}$$

Anharmonic PEL. To obtain the expressions for E_{IS} and E_{vib}, for a Gaussian and anharmonic PEL, we follow the same procedure outlined above. To do so, we first note that, in the presence of anharmonicities, the vibrational Helmholtz free energy is given by Eq. 17, which we rewrite as follows,

$$F_{\mathrm{vib}}(N,V,T,e_{\mathrm{IS}})\approx 3Nn_bk_BT\ln(\beta\hbar\omega_0)+k_BT\left[\mathcal{S}(N,V,T,e_{\mathrm{IS}})+\beta F^{\mathrm{anh}}_{\mathrm{vib}}(N,V,T)\right] \tag{S21}$$

The square bracket can be interpreted as an effective shape function,

$$\mathcal{S}_{\text{eff}}(N,V,T,e_{\text{IS}}) \equiv \mathcal{S}(N,V,T,e_{\text{IS}}) + \beta F_{\text{vib}}^{\text{anh}} \tag{S22}$$

that takes into account the corrections due to the anharmonicities in the PEL basins, about the corresponding IS. This means that the vibrational Helmholtz free energy is given by Eq. 11, substituting $\mathcal{S}_{\text{eff}}(N,V,T,e_{\text{IS}})$ for $\mathcal{S}(N,V,T,e_{\text{IS}})$,

$$F_{\text{vib}}(N,V,T) = 3Nn_b k_B T \ln(\beta\hbar\omega_0) + k_B T \mathcal{S}_{\text{eff}}(N,V,T,e_{\text{IS}}) \tag{S23}$$

Using Eq. S23, we can obtain E_{IS} and E_{vib}. The expression for E_{IS} (Eq. 18) follows from Eq. 8, with the configurational entropy given by Eq. 10 (Gaussian approximation), and the vibrational Helmholtz free energy given by Eq. S23. Substituting Eqs. 10 and S23 into Eq. 8, one can show that,

$$1 - k_B T \left[-\frac{(e_{\text{IS}} - E_0)}{\sigma^2} \right] + k_B T \left(\frac{\partial \mathcal{S}_{\text{eff}}}{\partial e_{\text{IS}}} \right)_{N,V,T} = 0 \quad \text{at} \quad e_{\text{IS}} = E_{\text{IS}} \tag{S24}$$

Using Eq. S22 in Eq. S24, it follows that,

$$E_{\text{IS}}(N,V,T) = E_0 - \sigma^2 (b + \beta) - \sigma^2 \left(\frac{\partial \beta F_{\text{vib}}^{\text{anh}}}{\partial e_{\text{IS}}} \right)_{N,V,T,e_{\text{IS}}=E_{\text{IS}}} \tag{S25}$$

which is identical to Eq. 18.

The expression for E_{vib} (Eq. 20) follows from the definition $E_{\text{vib}} \equiv E - E_{\text{IS}}$ and the thermodynamic expression $E = \left(\frac{\partial(\beta F)}{\partial \beta} \right)_{N,V}$. Since $F = -k_B T \ln Q$, it follows from Eq. 7 that the total energy is given by Eq. S13 even if anharmonicities are included. In the presence of anharmonicities, however, one must replace $\mathcal{S}(N,V,T,e_{\text{IS}})$ with $\mathcal{S}_{\text{eff}}(N,V,T,e_{\text{IS}})$ in the definition of F_{vib} [see Eqs. 11 and S23]. The same mathematical steps followed in the case of harmonic PELs [Eqs. S13–S20] can then be applied for anharmonic PELs. This implies that, in the presence of anharmonicities, Eq. S20 holds after substituting $\mathcal{S}_{\text{eff}}(N,V,T,e_{\text{IS}})$ for $\mathcal{S}(N,V,T,e_{\text{IS}})$. Therefore,

$$E_{\text{vib}} = 3Nn_b k_B T + \left(\frac{\partial \mathcal{S}_{\text{eff}}(N,V,T,E_{\text{IS}})}{\partial \beta} \right)_{N,V,E_{\text{IS}}} \tag{S26}$$

Using Eq. S22 in Eq. S26, one obtains the expression for E_{vib},

$$E_{\text{vib}}(N,V,T) = 3Nn_b k_B T + \left(\frac{\partial \mathcal{S}(N,V,T)}{\partial \beta} \right)_{N,V,E_{\text{IS}}} + \left(\frac{\partial (\beta F_{\text{vib}}^{\text{anh}}(N,V,T))}{\partial \beta} \right)_{N,V,E_{\text{IS}}} \tag{S27}$$

which is identical to Eq. 20.

VII. RELATIONSHIP BETWEEN THE TEMPERATURE-DEPENDENCE OF THE PEL OF A QUANTUM LIQUID AND THE SPRINGS POTENTIAL ENERGY OF THE CORRESPONDING RING-POLYMER SYSTEM

Here we show that, within the harmonic approximation of the PEL, the potential energy of the ring-polymer's springs,

$$E_{\rm sp}(N,V,T) \equiv \left\langle \sum_{i=1}^{N}\sum_{k=1}^{n_b} \frac{1}{2} k_i^{\rm sp} (\mathbf{r}_i^{k+1} - \mathbf{r}_i^{k})^2 \right\rangle_{N,V,T}$$

controls the temperature dependence of the PEL. Specifically, we show that

$$\left(\frac{\partial \mathcal{S}(N,V,T,e_{\rm IS})}{\partial \beta}\right)_{N,V,e_{\rm IS}=E_{\rm IS}} = -2\,E_{\rm sp}(N,V,T) \tag{S28}$$

To do so, we introduce an imaginary ring-polymer (IRP) system as follows. At a given temperature T, the ring-polymer system associated to the quantum liquid (briefly, 'the' RP system) is characterized by a spring constant $k^{\rm sp}(T) = m n_b/(\beta\hbar)^2$. We define the IRP system so its spring constant k_0 is T-independent and given by $k_0 \equiv k_{\rm sp}(T)$. Hence, while the PEL of the RP system (associated to the quantum liquid) varies with T, the PEL of the IRP does not. The IRP system is a truly classical system in the sense that its PEL, and hence its Hamiltonian, do not vary with T. Importantly, at the original temperature T, and only at this T, both IRP and the original RP share the same PEL, including the inherent structures available in the PEL.

Now, consider the evolution of the IRP system (with constant spring constant k_0) and the RP system associated to the quantum liquid [with spring constant $k^{\rm sp}(T)$] upon heating/cooling. The total energy of the original RP system is given by Eqs. 14 and 16 (harmonic approximation),

$$E(N,V,T) = E_{\rm IS}(N,V,T) \;+\; 3N\,n_b\,k_B T \;+\; \left(\frac{\partial \mathcal{S}(N,V,T,e_{\rm IS})}{\partial \beta}\right)_{N,V,e_{\rm IS}=E_{\rm IS}} \tag{S29}$$

Instead, for the IRP (T-independent PEL), the total energy (harmonic approximation) is given by

$$E^{\rm IRP} \;=\; E_{\rm IS}(N,V,T) \;+\; 3N\,n_b\,k_B T \tag{S30}$$

As shown in Ref. [9],

$$E(N,V,T) \;=\; E^{\rm IRP} \;-\; 2\,E^{\rm sp}.$$

Therefore, by substituting Eqs. S29 and S30 into this expression, one obtains Eq. S28.

---

[1] Ali Eltareb, Yang Zhou, Gustavo E. Lopez, and Nicolas Giovambattista. Potential energy landscape formalism for quantum molecular liquids. *Communications Chemistry*, 7(1):289, December 2024.

[2] Philip H. Handle and Francesco Sciortino. Potential energy landscape of TIP4P/2005 water. *The Journal of Chemical Physics*, 148(13):134505, April 2018.

[3] Raj K. Pathria and Paul D. Beale. *Statistical Mechanics*. Academic Press, an imprint of Elsevier, London San Diego Cambridge, MA Oxford, fourth edition edition, 2022.

[4] Yaakov Rosenfeld and Pedro Tarazona. Density functional theory and the asymptotic high density expansion of the free energy of classical solids and fluids. *Molecular Physics*, 95(2):141–150, October 1998.

[5] Srikanth Sastry. The relationship between fragility, configurational entropy and the potential energy landscape of glass-forming liquids. *Nature*, 409(6817):164–167, January 2001.

[6] Francesco Sciortino, Walter Kob, and Piero Tartaglia. Thermodynamics of supercooled liquids in the inherent-structure formalism: A case study. *Journal of Physics: Condensed Matter*, 12(29):6525–6534, July 2000.

[7] Mark E. Tuckerman. *Statistical Mechanics: Theory and Molecular Simulation*. Oxford Graduate Texts. Oxford University Press, Incorporated, Oxford, 2nd ed edition, 2023.

[8] Thomas E. Markland, Joseph A. Morrone, Bruce J. Berne, Kunimasa Miyazaki, Eran Rabani, and David R. Reichman. Quantum fluctuations can promote or inhibit glass formation. *Nature Physics*, 7(2):134–137, February 2011.

[9] Mark E. Tuckerman. *Statistical Mechanics: Theory and Molecular Simulation*. Oxford University Press, Oxford, 2010.
